# Nematicidal Activity of a Garlic Extract Formulation against the Grapevine Nematode *Xiphinema index*

**DOI:** 10.3390/plants12040739

**Published:** 2023-02-07

**Authors:** Trifone D’Addabbo, Edith Ladurner, Alberto Troccoli

**Affiliations:** 1 Institute for Sustainable Plant Protection—CNR, 70126 Bari, BA, Italy; 2 CBC Europe—Biogard Division, 24050 Grassobbio, BG, Italy

**Keywords:** *Xiphinema index*, grapevine, sustainable control, garlic extract

## Abstract

The nematicidal activity of garlic extracts is known on root–knot nematodes but never investigated on the grapevine nematode *Xiphinema index*. In this study, the nematicidal activity of a commercial garlic extract formulate (GEF) was assessed on *X. index*, both in vitro and in a pot assay. In the in vitro assays, mixed specimens of *X. index* were exposed to a 0–4 mL L^−1^ range of GEF concentrations, checking nematode immotility and mortality after 2, 4 or 8 h. In the experiments on potted grapevines, plants cultivated in soil infested by *X. index* were irrigated twice at a 15-day interval with 0.05, 0.2 and 0.5 mL L^−1^ solutions of GEF, including nontreated soil as a control. An almost complete mortality of *X. index* specimens occurred after a 2 h exposure to a 2 mL L^−1^ GEF concentration, while an 8 h exposure to even the 0.0312 and 0.0156 mL L^−1^ solutions resulted in about 50% and 30% mortality, respectively. Soil treatment with a 0.5 mL L^−1^ GEF solution significantly reduced the population of *X. index* and increased the grapevine root growth compared to nontreated soil or soil treated with the lower dosages. Results of this study indicated that garlic-based nematicides could be an effective tool for *X. index* management in organic and integrated vineyards.

## 1. Introduction

The parasitism of the dagger nematode, *Xiphinema index* Thorne et Allen can reduce grapevine yield and quality, causing severe economic losses in vineyards throughout the world [1,2]. Yield losses are mainly due to *X. index*’s role as vector of the grapevine fanleaf virus, as direct plant damages are limited to stubby roots with terminal swellings, rarely affecting grapevine growth and yield performance [3]. Over the last decades of the past century, control of *X. index* in vineyards mainly relied on pre–planting soil treatments with chemical fumigants as methyl bromide and 1,3-dichloropropene, which are currently withdrawn from the market because of their high environmental toxicity [4]. Poor availability of effective synthetic nematicides has created a strong desire for the search of alternative control tools, such as organic amendments [5], green manures [6], biofumigation [7] and biocontrol agents [8]. 

Based on the presence of nematicidal compounds in a large variety of plants [9], plant–derived nematicide products could be an additional tool for the environmentally safe management of *X. index*. Previous studies of our research group documented a high sensitivity of *X. index* to various biocidal plant secondary metabolites, such as saponins, prosapogenins and sapogenins from different *Medicago* species [10], caffeic acid, chlorogenic acid and artemisinin from *Artemisia annua* L [11], or essential oils from aromatic and medicinal plants [12]. Moreover, an antagonistic effect on *X. index* was also documented for the extracts or biomasses of other plant species, such as *Brassica napus* L., *B. juncea* Czern. & Coss, *Chenopodium ambrosioides* L. and *Ruta graveolens* L. [13,14].

Garlic (*Allium sativum* L., Amaryllidaceae family) has been documented for a wide spectrum of biological effects, including antibacterial, antifungal and nematicidal activities, attributed to the presence of a wide range of organosulfur compounds [15,16]. In particular, the most bioactive class of garlic compounds are the diallyl polysulfide compounds with one to six sulfur atoms in a linear chain, deriving from the enzymatic hydrolyzation of sulfur–containing amino acids through the formation of the unstable compound allicin [17]. The nematicidal activity of garlic extracts or other garlic formulations has been documented, though at different levels of efficacy, on root–knot nematodes [15,18,19,20]. More recently, granular or emulsifiable formulations of garlic extracts rich in polysulfides have been proved for an effective control of the root–knot nematode *Meloidogyne incognita* (Kofoid et White) Chitwood on tomato and other vegetable crops [21,22,23]. These formulations could represent a potential tool also for the control of *X. index* in vineyards but, to the best of our knowledge, there is no current information on their efficacy on this phytonematode species. Based on this lack of information, this study aimed to investigate the effects of a commercial garlic extract formulate (GEF) against *X. index*, both with in vitro conditions and in soil.

## 2. Results

### 2.1. In Vitro Toxicity

An almost complete mortality of *X. index* specimens occurred already after a 2 h exposure at the 4 and 2 mL L^−1^ concentrations of GEF, though more than 80% and 60% mortality rates were also found at the 1.0 and 0.5 mL L^−1^ concentrations, respectively (Table 1).

At the same exposure time, nematode motility and mortality was not or poorly affected by GEF concentrations ≤0.125 mL L^−1^. Moreover, both immotility and mortality rates resulted in no statistical differences with the 0–0.0625 mL L^−1^ concentrations, while significant differences occurred among the other concentrations. After a 4 h treatment, a complete *X. index* mortality occurred in the 0.5–4 mL L^−1^ concentrations and 90% and 73% of treated specimens were immobilized or killed, respectively, even in a 0.250 mL L^−1^ product solution.

The 8 h exposure resulted in almost 50% and 25% *X. index* mortality rates even at the 0.0312 and 0.0156 mL L^−1^ GEF concentrations, respectively, while nematode immobilization was almost complete after an immersion in the 0.125 mL L^−1^ solution for the same time. At both the 4 and 8 h exposure, immotility and mortality rates significantly increased only in the range of 0–0.250 mL L^−1^ concentrations, as equal to 100% in solutions ≥0.5 mL L^−1^. Data from the probit analysis indicated that 0.26 and 0.32 mL L^−1^ GEF concentrations were sufficient to immobilize (IC_50_) or to kill (LC_50_), respectively, 50% of the exposed *X. index* specimens within 2 h, while these values dropped to 0.027 and 0.044 mL L^−1^ for the 8 h treatment.

### 2.2. Activity in Soil

**Figure 1:**
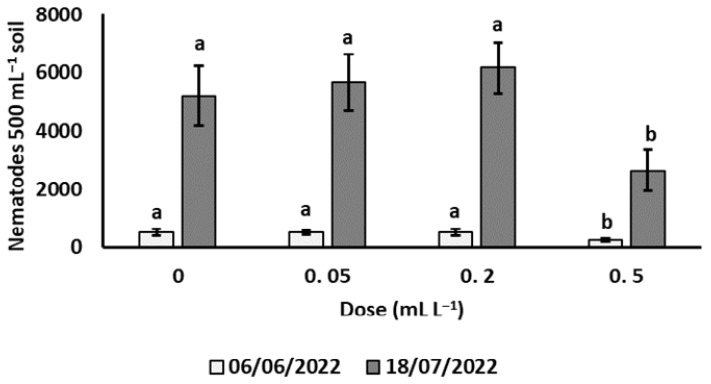
Effect of soil treatments with garlic extract formulation on soil population density of *Xiphinema index* on potted grapevine. Values are means of seven replicates ± standard errors. At each sampling date, bars marked by the same letters are not significantly different according to the Least Significant Difference Test (*p* ≤ 0.05).

Compared to the nontreated control, soil population density of *X. index* was significantly reduced only by the 0.5 mL L^−1^ treatment of GEF at both sampling dates, −52.8 and −49.4%, respectively (Figure 1). 

No significant effect on the *X. index* population was provided by soil treatments with 0.05 and 0.2 mL L^−1^ GEF solutions. The suppressive effect of the garlic product persisted for about one month after the second treatment, as nematode population rapidly increased in all pots until the end of the experiment, due to the maintenance of grapevine plants at a favourable greenhouse temperature (25 ± 2 °C). However, the *X. index* reproduction rates in the interval between the two sampling dates remained slightly lower in soil treated with 0.5 mL L^−1^ of GEF than in soil not treated or treated with the other two dosages (9.8 vs. 10.8, 11.9 and 10.5, respectively). 

**Figure 2:**
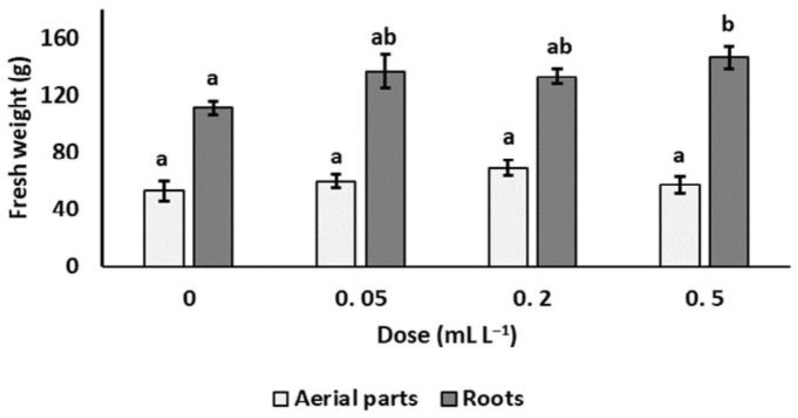
Effect of soil treatments with garlic extract formulation on the potted grapevine plants in soil infested with *Xiphinema index*. Values are means of seven replicates ± standard errors. For each plant part, bars marked by the same letters are not significantly different according to the Least Significant Difference Test (*p* ≤ 0.05).

Weight of the aboveground biomass of grapevine plants did not significantly differ among the treatments, while a significant increase of root biomass weight (+37.1%) was recorded in soil treated with the 0.5 mL L^−1^ GEF solution compared to the control (Figure 2).

## 3. Discussion

Data from the in vitro assay proved a strong activity of GEF on *X. index*, as 1.0 and 0.25 mL L^−1^ concentrations were sufficient to kill more than 90% of the *X. index* specimens within 2 and 8 h, respectively. However, a preliminary microscopical observation (data not showed) indicated that the toxic effects of GEF on the *X. index* specimens started even earlier than the first 2 h check reported in the study. The *X. index* sensitivity to GEF was higher than that of the root–knot nematode *M. incognita*, as the values of LC_50_ calculated in our in vitro experiments (0.32, 0.096 and 0.044 mL L^−1^ after 2, 4 and 8 h, respectively) were much lower than those (0.8 mg mL^−1^ after 96 h) reported for *M. incognita* in previous assays with a granular formulation of the same product tested in this study [21]. The higher sensitivity of *X. index* was in agreement with our previous studies on other phytochemicals, such as sesquiterpenes from *A. annua* or monoterpenes from various essential oils [13,14], and could be reasonably attributed to the different anatomy and feeding behavior of the *X. index* and *Meloidogyne* species [24,25]. 

This study is the first report of an in vitro toxicity to *X. index* of GEF, while the activity of raw or formulated garlic extracts has been already documented on root–knot nematode species. The study of Eder et al. [21] reported a strong nematicidal activity on *M. incognita* second stage juveniles (J2) by solutions of a granular garlic extract formulation containing the same technical grade material as the liquid formulation tested in our experiments. In earlier studies, Sukul et al. [18] reported a 61% mortality of *M. incognita* J2, even after a 5 min exposure to a garlic ethanol extract, as well as a garlic aqueous extract which killed 100% of *M. incognita* J2 within 72 h [19]. Additional studies by the same authors described a strong reduction of *M. incognita* egg hatching and J2 mortality by treatments with allicin, i.e., the precursor of polysulfides contained in the GEF tested in this study [20].

The strong in vitro activity of GEF on *X. index* was also confirmed in soil, as the product was able to significantly suppress the *X. index* population density at a 0.5 mL L^−1^ concentration. This result is in good agreement with data from the in vitro assays, in which the 0.5 mL L^−1^ GEF concentration was the lowest to provide a complete nematode mortality after a 4 h exposure. The 0.5 mL L^−1^ rate used in the pots corresponds to a field dosage of 0.5 L 1000 L^−1^ water, a dose easily appliable within the conventional agronomical techniques. In addition, this dosage also showed a longer residual effect compared to the other two tested dosages, as proved by the lower nematode reproduction rate after the end of the treatments. As for the in vitro assays, this is the first report of the suppressiveness of a garlic–derived product to the soil population density of *X. index*, while previous literature data have documented a strong nematicidal activity of garlic products in soil infested by *M. incognita*. In the study by Sukul et al. [18], soil treatments with an aqueous extract of garlic effectively reduced *M. incognita* infestation on okra (*Abelmoschus esculentus* L.). In addition, a root–dip treatment of tomato seedlings with allicin solutions significantly reduced the penetration of tomato roots by *M. incognita* J2 [20]. The granular garlic extract product recently tested by Eder et al. [21] showed a significant suppressiveness on *M. incognita* infestation on potted tomato, as well as previously ensured a satisfactory root–knot nematode control in field experiments on various horticultural crops [22]. Inhibitory effects on soil population density of *M. incognita* and other phytonematode genera (*Rotylenchus* spp., *Pratylenchus* spp., *Helicotylenchus* spp. and *Tylenchorhynchus* spp.) were also achieved by intercropping garlic with grapevine or vegetable crops [26,27]. However, the evaluation of the nematicidal performance of garlic products should also take into consideration the soil composition, as movement of diallyl disulfide can largely vary among sandy loam, fine sand or silty clay loam soil textures [28].

The mode of action of diallyl polysulfides, i.e., the main active compounds of garlic–derived nematicides, is still unclear, though their multi–site activity should probably be hypothesized [29]. Chatterji et al. [30] observed a DNA damage and/or cell apoptosis following the formation of reactive oxygen species by polysulfide compounds. Due to the metal binding capacity of diallyl polysulfides, a disturbance of metal homeostasis has been also suggested [31]. The GEF investigated in this study did not show any phytotoxic effects on grapevine plants, not affecting aboveground plant biomass and even improving root system development at the highest tested dosage. In good agreement with our results, no side effects on tomato plants were reported also for the granular garlic extract product tested by Eder et al. [21].

## 4. Materials and Methods

### 4.1. Nematodes

The population of *X. index* was collected from an infested vineyard at Noicattaro (province of Bari, Southern Italy) and reared on potted grapevine plants arranged outdoor under a shade. The specimens of *X. index* were extracted from the soil by repeated decanting and sieving on 90 μ sieves followed by an overnight incubation in 75 μ Oostenbrink’s sieves placed within 12 cm diameter Petri dishes filled with distilled water [32].

### 4.2. Bioassay In Vitro

Batches of 50 specimens at different developmental stages (juveniles and adults) of *X. index* were placed in ridged 5 cm plastic Petri dishes (4 mL vol) containing 2 mL of distilled water. The Petri dishes were then added with 2 mL of 0.0312, 0.0625, 0.125, 0.25, 0.5, 1, 2, 4 and 8 mL L^−1^ water solutions of a commercial nematicide (Nemguard^®^ SC, manufacturer: Ecospray Ltd., Suffolk, UK; distributor: CBC (Europe) S.r.l., Grassobbio (Bergamo), IT) formulated as a suspension concentrate containing 100% garlic extract (GEF). Therefore, the final test concentrations were 0.0156, 0.0312, 0.0625, 0.125, 0.25, 0.5, 1, 2, and 4 mL L^−1^. The Petri dishes were then capped and sealed with parafilm to avoid any dispersal of the active product volatiles (polysulphides). The *X. index* specimens were exposed to each test solution for 2, 4, or 8 h. Four replicates were provided for each concentration × exposure time combination, including dishes containing only distilled water as a control. At the end of each exposure time, each replicate was observed under a stereo–microscope and checked for nematode motility/immotility. The mortality of immotile *X. index* specimens were microscopically assessed after their stimulation with a drop of NaOH [33]. 

Nematode immotility and mortality rates were calculated by the Abbott’s formula m = 100 × (1 − nt/nc), where m = percent immotility/mortality; nt = number of motile/viable nematodes after the treatment; and nc = number of motile/viable nematodes in water.

The experiment was run twice, pooling data from the two experimental runs.

### 4.3. Experiment In Soil

The rooted grapevine cuttings (rootstock 110R) were transplanted in 2 L clay pots filled with a sterilized sandy soil in November 2021. At the spring vegetative restart on March 2022, each pot was inoculated with 120 mixed specimens (adults and juveniles) of *X. index*. On 26 April 2022 the soil of each pot was at first moistened with 100 mL of water, then treated with 200 mL of 0.05, 0.2 and 0.5 mL L^−1^ GEF solutions and finally added with a 50 mL volume of water. These treatments were repeated after two weeks (10 May 2022). The control was represented by nontreated soil. Seven replicates were provided for each treatment and control. The pots were arranged on the benches of a glasshouse maintained at a constant temperature of 25 ± 2 °C. The population density of *X. index* was monitored on 6 June and 18 July 2022 by extracting nematodes from a 500 mL soil sample collected from each pot, according to the same decanting and sieving extraction technique described above [32]. After the second soil sampling, grapevine plants were uprooted and the weight of the aerial parts and roots was recorded. 

### 4.4. Statistical Analysis

All data were statistically analyzed by ANOVA and means were separated by the Least Significant Difference Test (*p* ≤ 0.05), using the software PlotIT 3.2 (Scientific Programming Enterprises, Haslett, MI, USA). Data from the in vitro assays were also subjected to probit analysis to calculate the concentrations needed to immobilize (IC_50_) or to kill (LC_50_) 50% of the exposed *X. index* specimens. 

## 5. Conclusions

The tested GEF showed a potent in vitro activity on *X. index* also when applied at very low concentrations. As previously remarked, the product was also effective on *X. index* when applied to soil at 0.5 mL L^−1^, corresponding to a 0.5 L 1000 L^−1^ water field rate, i.e., a dosage compatible with conventional agronomical techniques in vineyards. However, additional studies should be carried out to verify the nematicidal effectiveness, technical feasibility and potential side effects on grapevine plant health at higher dosages. Soil treatments with GEF did not show negative effects on plant growth and even slightly stimulated root growth, also ensuring a plant–safe application during the grapevine crop cycle. 

The liquid formulation of GEF allows for an easy and uniform distribution in vineyards without interfering with grapevine cultural practices or adversely affecting other agronomical techniques, such as green manures, biofumigation or organic amendments, which are often suggested for preventing or reducing the diffusion of *X. index* but are technically difficult to correctly apply in vineyards. 

The GEF investigated in this study, as well as other garlic–derived nematicides, could represent a useful tool for the management of *X. index* in both organic and integrated viticulture, especially in consideration of the lack of synthetic or biological nematicides registered for grapevines. In addition to the assessment of the nematicidal effectiveness of higher dosages, synergistic effects derived from the combination of garlic products with other nonchemical control tools, such as biocontrol agents, cover crops and organic amendments, should be also investigated. Finally, in accordance with the literature reports, the variability of the efficacy of garlic products in the presence of different soil textures should be also verified.

## Figures and Tables

**Table 1:** Effect of 2-, 4- and 8-h in vitro exposure to a 0–4 mL L^−1^ range of concentrations of a garlic extract formulate on motility and mortality of mixed–age specimens of *Xiphinema index* ^1^.

mL L^−1^	2 h				4 h				8 h			
	Immotility		Mortality		Immotility		Mortality		Immotility		Mortality	
0	0.0 ± 0	a ^2^	0.3 ± 0.3	a	0.3 ± 0.3	a	0.4 ± 0.4	a	0.4 ± 0.4	a	0.4 ± 0.4	a
0.0156	3.2 ± 0.6	a	2.8 ± 0.5	a	18.8 ± 1.0	b	13.0 ± 0.9	b	35.6 ± 0.5	b	25.1 ± 2.0	b
0.0312	5.3 ± 0.2	a	4.7 ± 0.3	a	31.7 ± 1.6	c	27.8 ± 0.9	c	56.0 ± 1.0	c	46.9 ± 2.0	c
0.0625	6.8 ± 0.7	a	5.0 ± 0.9	a	37.8 ± 1.5	d	35.9 ± 1.1	d	70.1 ± 3.0	d	57.2 ± 1.4	d
0.125	14.5 ± 0.2	b	13.6 ± 0.4	b	46.9 ± 0.9	e	58.3 ± 1.2	e	94.1 ± 3.6	e	66.7 ± 1.7	e
0.250	35.6 ± 1.8	c	28.1 ± 1.6	c	89.7 ± 1.7	f	72.6 ± 0.9	f	99.1 ± 0.7	f	91.0 ± 1.0	f
0.5	69.9 ± 5.4	d	61.9 ± 4.3	d	100 ± 0	g	100 ± 0	g	100 ± 0	f	100 ± 0	g
1.0	90.0 ± 1.7	e	83.6 ± 1.3	e	100 ± 0	g	100 ± 0	g	100 ± 0	f	100 ± 0	g
2.0	97.1 ± 1.5	f	97.1 ± 1.5	f	100 ± 0	g	100 ± 0	g	100 ± 0	f	100 ± 0	g
4.0	100 ± 0	f	100 ± 0.0	f	100 ± 0	g	100 ± 0	g	100 ± 0	f	100 ± 0	g
IC50	0.26	-	-	-	0.076	-	-	-	0.027	-	-	-
LC50	-	-	0.32	-	-	-	0.096	-	-	-	0.044	-

^1^ Values are means of four replicates ± standard error; ^2^ Means followed by the same letter on the same column are not significantly different according to the Least Significant Difference Test (*p* ≤ 0.05).

## Data Availability

All data are available upon request from the corresponding author.

## References

[1] Jones J.T., Haegeman A., Danchin E.G.J., Gaur H.S., Helder J., Jones M.G.K., Kikuchi T., Manzanilla–López R., Palomares–Rius J.E., Wesemael W.M.L. (2013). Top 10 plant–parasitic nematodes in molecular plant pathology. Mol. Plant Pathol..

[2] Van Zyl S., Vivier M.A., Walker M.A. (2012). *Xiphinema index* and its relationship to grapevines: A review. S. Afr. J. Enol. Vitic..

[3] Andret–Link P., Laporte C., Valat L., Ritzenthale C., Demangreat G., Vigne E., Laval V., Pfeiffer P., Stussi–Garaud C., Fuchs M. (2004). Grapevine fanleaf virus: Still a major threat to the grapevine industry. J. Plant Pathol..

[4] Oka Y., Saroya Y. (2019). Effect of fluensulfone and fluopyram on the mobility and infection of second-stage juveniles of *Meloidogyne incognita* and *M. javanica*. Pest Manag. Sci..

[5] D’Addabbo T., Sasanelli N., Coiro M.I. (1999). Suppression of *Xiphinema index* by olive ansd grape pomace. Nematol. Medit..

[6] Villate L., Morin E., Demangeat G., Van Helden M., Esmenjaud D. (2012). Control of *Xiphinema index* populations by fallow plants under greenhouse and field conditions. Phytopathology.

[7] Bello A., Arias M., López–Pérez J.A., García–Álvarez A., Fresno J., Escuer M., Arcos S.C., Lacasa A., Sanz R., Gómez P. (2004). Biofumigation, fallow, and nematode management in vineyard replant. Nematropica.

[8] Aballay E., Mårtensson A., Persson P. (2011). Screening of rhizosphere bacteria from grapevine for their suppressive effect on *Xiphinema index* Thorne & Allen on in vitro grape plants. Plant Soil.

[9] D’Addabbo T., Laquale S., Lovelli S., Candido V., Avato P. (2014). Biocide plants as a sustainable tool for the control of pests and pathogens in vegetable cropping systems. Ital. J. Agron..

[10] Argentieri M.P., D’Addabbo T., Tava A., Agostinelli A., Jurzysta M., Avato P. (2008). Evaluation of nematicidal properties of saponins from Medicago spp. Eur. J. Plant Pathol..

[11] D’Addabbo T., Carbonara T., Argentieri M.P., Radicci V., Leonetti P., Villanova L., Avato P. (2013). Nematicidal potential of *Artemisia annua* and its main metabolites. Eur. J. Plant Pathol..

[12] Avato P., Laquale S., Argentieri M.P., Lamiri A., Radicci V., D’Addabbo T. (2017). Nematicidal activity of essential oils from aromatic plants of Morocco. J. Pest Sci..

[13] Aballay E., Sepúlveda R., Insunza V. (2004). Evaluation of five nematode–antagonistic plants used as green manure to control *Xiphinema index* Thorne et Allen on *Vitis vinifera* L. Nematropica.

[14] Sasanelli N. (1992). Nematicidal activity of aqueous extracts from leaves of *Ruta graveolens* on *Xiphinema index*. Nematol. Medit..

[15] Anwar A., Gould E., Tinson R., Groom M., Hamilton C.J. (2017). Think yellow and keep green–role of sulfanes from garlic in agriculture. Antioxidants.

[16] Münchberg U., Anwar A., Mecklenburg S., Jacob C. (2007). Polysulfides as biologically active ingredients of garlic. Org. Biomol. Chem..

[17] Block E., Naganathan S., Putman D., Zhao S.H. (1993). Organosulfur chemistry of garlic and onion—Recent results. Pure Appl. Chem..

[18] Sukul N.C., Das P.K., De G.C. (1974). Nematicidal action of some edible crops. Nematologica.

[19] Gupta R., Sharma N.K., Kaur D. (1985). Efficacy of garlic as nematicide against *Meloidogyne incognita* (Kofoid and White, 1919) Chitwood (1949). Ind. J. Nematol..

[20] Gupta R., Sharma N.K. (1993). A study of the nematicidal activity of allicin–an active principle in garlic, *Allium sativum* L., against root–knot nematode, *Meloidogyne incognita* (Kofoid and White, 1919) Chitwood, 1949. Int. J. Pest. Manag..

[21] Eder R., Consoli E., Krauss J., Dahlin P. (2021). Polysulfides applied as formulated garlic extract to protect tomato plants against the root–knot nematode *Meloidogyne incognita*. Plants.

[22] Ladurner E., Benuzzi M., Fiorentini F., Lucchi A. (2014). Efficacy of NemGuard® granules, a new nematicide based on garlic extract for the control of root–knot nematodes on horticultural crops. Proceedings of the Atti Giornate Fitopatologiche, Chianciano Terme.

[23] Anwar A., Groom M., Sadler–Bridge D. (2009). Garlic from Nature’s ancient food to nematicide. Pestic. News.

[24] Davies K.G., Curtis R.H.C. (2011). Cuticle surface coat of plant–parasitic nematodes. Ann. Rev. Phytopathol..

[25] Yeats G.W., Bongers T., De Goede R.G.M., Freckman D.W., Georgieva S.S. (1993). Feeding habits in soil nematode families and genera—An outline for soil ecologists. J. Nematol..

[26] Samy F.M., El–Ashry R.M.A., Zyada H.G. (2021). Evaluation the effect of intercropping garlic with grapevines on productivity, phytoremediation, competitive indices and plant parasitic nematode community. J. Plant Prod..

[27] Ameen H.H. (1996). Influence of garlic *Allium sativum* on populations of *Rotylenchulus reniformis* and *Meloidogyne incognita* infecting cowpea and tomato. Al–Azhar J. Agric. Res..

[28] López–Serna R., Ernst F., Wu L. (2016). Analysis of cinnamaldehyde and diallyl disulfide as eco–pesticides in soils of different textures–a laboratory–scale mobility study. J. Soil Sed..

[29] Sparks T.C., Crossthwaite A.J., Nauen R., Banba S., Cordova D., Earley F., Ebbinghaus–Kintscher U., Fujioka S., Hirao A., Karmon D. (2020). Insecticides, biologics and nematicides: Updates to IRAC’s mode of action classification—A tool for resistance management. Pestic. Biochem. Physiol..

[30] Chatterji T., Keerthi K., Gates K.S. (2005). Generation of reactive oxygen species by a persulfide (BnSSH). Bioorg. Med. Chem. Lett..

[31] Anwar A., Groom M., Arbach M., Hamilton C.J., Jacob C., Kirsch G., Slusarenko A., Winyard P.G., Burkholz T. (2014). How to turn the chemistry of garlic into a ‘botanical’pesticide. Recent Advances in Redox Active Plant and Microbial Products.

[32] van Bezooijen J. (2006). Methods and Techniques for Nematology.

[33] Chen S.Y., Dickson D.W. (2000). A technique for determining live second–stage juveniles of *Heterodera glycines*. J. Nematol..

